# Applications of transformer-based language models in bioinformatics: a survey

**DOI:** 10.1093/bioadv/vbad001

**Published:** 2023-01-11

**Authors:** Shuang Zhang, Rui Fan, Yuti Liu, Shuang Chen, Qiao Liu, Wanwen Zeng

**Affiliations:** College of Software, Nankai University, Tianjin 300350, China; College of Software, Nankai University, Tianjin 300350, China; College of Software, Nankai University, Tianjin 300350, China; College of Software, Nankai University, Tianjin 300350, China; Department of Statistics, Stanford University, Stanford, CA 94305, USA; College of Software, Nankai University, Tianjin 300350, China; Department of Statistics, Stanford University, Stanford, CA 94305, USA

## Abstract

**Summary:**

The transformer-based language models, including vanilla transformer, BERT and GPT-3, have achieved revolutionary breakthroughs in the field of natural language processing (NLP). Since there are inherent similarities between various biological sequences and natural languages, the remarkable interpretability and adaptability of these models have prompted a new wave of their application in bioinformatics research. To provide a timely and comprehensive review, we introduce key developments of transformer-based language models by describing the detailed structure of transformers and summarize their contribution to a wide range of bioinformatics research from basic sequence analysis to drug discovery. While transformer-based applications in bioinformatics are diverse and multifaceted, we identify and discuss the common challenges, including heterogeneity of training data, computational expense and model interpretability, and opportunities in the context of bioinformatics research. We hope that the broader community of NLP researchers, bioinformaticians and biologists will be brought together to foster future research and development in transformer-based language models, and inspire novel bioinformatics applications that are unattainable by traditional methods.

**Supplementary information:**

Supplementary data are available at *Bioinformatics Advances* online.

## 1 Introduction

Bioinformatics, an interdisciplinary research field, has become one of the most influential areas of life science research in a profound way. It is characterized by the demand to develop and utilize computational tools and methods to analyze huge amounts of biomedical data and translate them into knowledge for developing downstream applications.

**Fig. 1.**
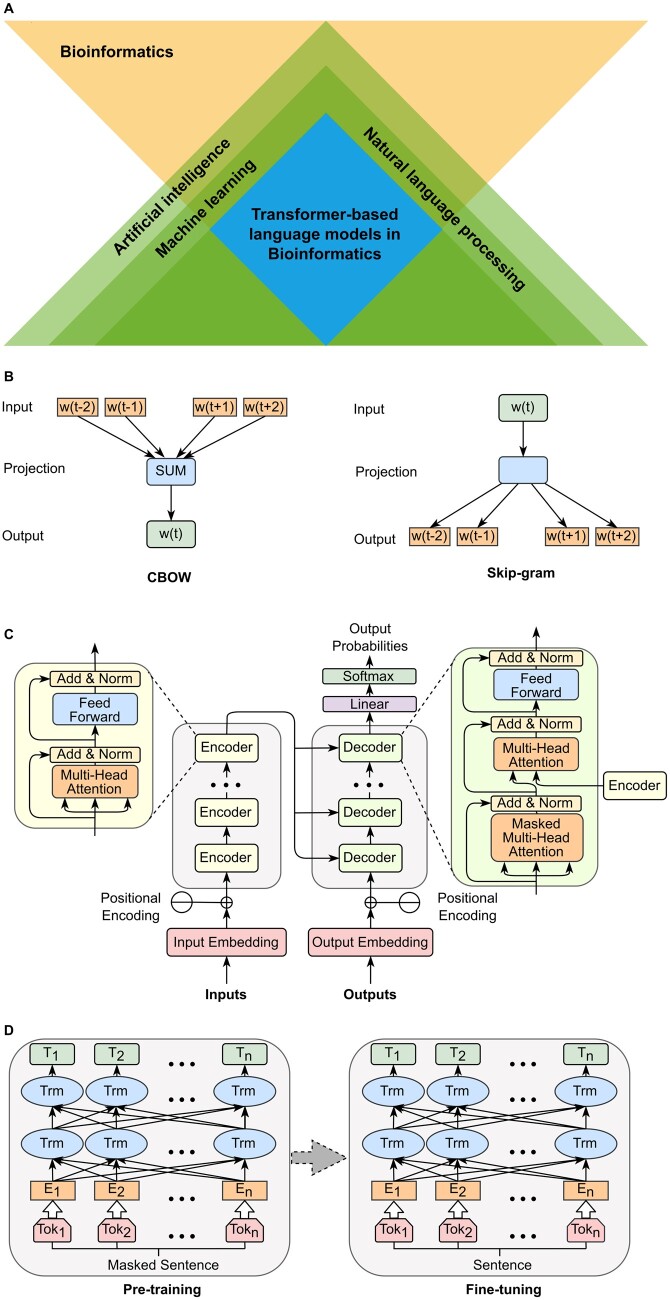
The focus of this review article and some classic language models frameworks. (**A**) Relationships of artificial intelligence, machine learning, natural language processing, transformer-based language models and bioinformatics. The blue square denotes the focal point of this review article. (**B**) Two common models in Word2Vec: CBOW (Continuous Bag-of-Words Model) and Skip-gram (Continuous Skip-gram Model). (**C**) The structure of transformer model. (**D**) The structure of BERT model

In recent years, natural language processing (NLP) (Nadkarni *et al.*, 2011; Supplementary Table S1), a branch of artificial intelligence, has been increasingly showing a substantial impact in bioinformatics research fields (Han and Kwoh, 2019), ranging from DNA/RNA sequence analysis to computational biology (Iuchi *et al.*, 2021; Zeng *et al.*, 2018). Specifically, NLP technologies, with the aim to grant computers the ability to understand words and texts from human beings (Tsujii, 2021), have the potential power to also understand biological languages. Language models enable computers to analyze the patterns of human language by predicting words (Adel *et al.*, 2018) (Fig. 1A) and are becoming one of the core technologies for many NLP tasks, including sentiment analysis (Schouten and Frasincar, 2016), machine translation (Bahdanau *et al.*, 2016) and text summarization (Nenkova and McKeown, 2012). The history of leveraging the power of neural networks (NNs) (Walczak and Cerpa, 2003) in NLP tasks can be tracked back two decades (Bengio *et al.*, 2003), where a series of word embedding technologies were proposed to provide a novel representation of text and achieved superior results (Blacoe and Lapata, 2012; Turian *et al.*, 2010). For example, Word2Vec (Le and Mikolov, 2014; Mikolov *et al.*, 2013a, b), which maps one-hot word vectors to distributed word vectors using a shallow neural network, is one of the most representative models. Word2vec can utilize either of two types of model architecture to produce these distributed representations of words: continuous bag-of-words (CBOW) or continuous skip-gram. CBOW predicts the current word based on the context while skip-gram predicts surrounding words given the current word (Fig. 1B). With the rapid development of deep learning technologies (LeCun *et al.*, 2015), language models in NLP have continuously made significant breakthroughs: conventional RNN-based models, including Bi-RNN (Schuster and Paliwal, 1997), LSTM (Hochreiter and Schmidhuber, 1997) and GRU (Cho *et al.*, 2014), attempt to encode the entire sequence into a finite length vector without paying more attention to those important works. Although these RNN-based models are able to learn long-term dependency, they greatly suffer from vanishing gradient and low-efficiency problems as they sequentially process all past states and compress contextual information into a bottleneck with long input sequences (Bengio *et al.*, 1994; Pascanu *et al.*, 2013). For example, Seq2Seq (Sutskever *et al.*, 2014), the first encoder–decoder model in machine translation tasks, supports variable-length inputs and outputs but is still limited by its infrastructure LSTM. The Transformer (Vaswani *et al.*, 2017) model was then developed by Google, which completely abandoned RNN-based network structures, and only used the multi-head attention mechanism (Fig. 1C). Transformer does not rely on the past hidden states to capture the dependency on the previous words. Instead, transformer processes a sentence as a whole to allow for parallel computing and alleviates the vanishing gradient and performance degradation caused by long-term dependency. In this review article, we will focus on transformer-based language models.

In general, transformer-based language models fall into two categories: scratch-trained models and pre-trained models. The scratch-trained models directly train all model parameters from the beginning using task-specific datasets and often require many iterations to fully converge. For example, Transformer-XL (Dai *et al.*, 2019) uses relative positional encoding and segmented RNN mechanism to model long text; Sparse Transformers (Zhao *et al.*, 2019) uses only a small number of tokens in the computation of attention distribution to improve the concentration of attention mechanism; Reformer (Kitaev *et al.*, 2020) addresses the resource-hungry problem of the transformer by replacing dot-product attention and using reversible residual layers; Longformer (Beltagy *et al.*, 2020) proposes sliding windows, dilated sliding windows and global attention strategies to reduce the complexity of the model. On the other hand, transformer-based pre-trained models are trained from large amounts of unlabeled data and then fine-tuned for specific tasks. Pre-training learns general information from unlabeled data, speeds up the convergence rate of the target tasks and usually has better generalization than training parameters from scratch (Han *et al.*, 2021). For example, GPT-X (Brown *et al.*, 2020; Radford and Narasimhan, 2018; Radford *et al.*, 2019) proposes unsupervised pre-training and supervised fine-tuning for the first time; BERT (Devlin *et al.*, 2019) utilizes bi-directional transformers and mask mechanism (Fig. 1D) to achieve a deeper understanding of context than GPT; RoBERTa (Liu *et al.*, 2019b) uses dynamic masking and has a significant improvement over BERT in terms of model size and arithmetic power; XLNet (Yang *et al.*, 2019b), which is based on the Transformer-XL architecture, further introduces permutation language modeling as an improved training method; ERNIE (Zhang et al., 2019) adopts a continual learning mechanism, which consists of two parts: continual construction of pre-training tasks and incremental multi-task learning; ALBERT (Lan *et al.*, 2020) is a mini-model using cross-layer parameter sharing and paragraph continuity tasks; T5 (Raffel *et al.*, 2020) is a generic framework that converts all NLP tasks into Text-to-Text format. These two types of transformer-based language models show their strength in addressing key challenges and have become a quintessential choice in almost all NLP tasks (Casola *et al.*, 2022; Chaudhari *et al.*, 2021). These breakthroughs in methodologies and technologies have revolutionized the field of NLP, thus bringing the thoughts of applications in biological and biomedical research.

Although there are reviews of transformers in the general domain (Kalyan *et al.*, 2021b; Lin *et al.*, 2022; Qiu *et al.*, 2020) and a survey of transformer-based biomedical pre-trained language models (Kalyan *et al.*, 2021a), the applications of transformer-based language models in the latest bioinformatics research, such as spatial transcriptomics and multi-omics, have not yet been documented. In this review, we provide a comprehensive viewpoint of facilitating research in the field of NLP and the applications of transformers in bioinformatics. We revisit the basics of transformer-based language models, summarize the latest developments in the transformer-based language models and then review the applications of transformers in bioinformatics and biomedical downstream tasks such as sequence analysis, gene expression, proteomics, spatial transcriptomics, etc. Last but not least, we discuss the future challenges and opportunities in using and understanding multi-omics high-throughput sequencing data. We hope that transformer-based language models not only benefit the computer science community but also the broader community of bioinformaticians and biologists, and further provide insights for future bioinformatics research across multiple disciplines that are unattainable by traditional methods.

## 2 Basics of transformer-based language models

Language models are trained in a self-supervised fashion (Liu *et al.*, 2023). Compared to supervised learning (Hastie *et al.*, 2009), which usually needs human annotations, language models could use massive amounts of unannotated corpora from the internet, books, etc. Language models either take the next word as a natural label for the context in a sentence or artificially mask a known word and then predict it (Petroni *et al.*, 2019). The paradigm that uses the unstructured data itself to generate labels (e.g. the next word or the masked word in language models) and train supervised models (language models) to predict labels is called ‘self-supervised learning’ (Howard and Ruder, 2018). Specifically, because of their parallelism and the ability to extract correlation across the whole sequences, transformer-based models achieve state-of-the-art (SOTA) performance in a variety of important tasks such as machine translation and question answering (QA) (Pundge *et al.*, 2016). Since there are high similarities between human language and bioinformatics sequence data, transformer-based models are becoming one of the most promising models to tackle the sequence-based problems in bioinformatics (Ofer *et al.*, 2021).

The vanilla transformer model can be divided into two parts: encoder and decoder, which have similar basic architectures composed of a stack of identical blocks (Vaswani *et al.*, 2017). Each block consists of two kinds of sub-layers: the multi-head attention sub-layer and the position-wise feed-forward sub-layer. Both kinds of sub-layers are followed by layer normalization. A residual connection around every sub-layer will be applied in each block to speed up the training process. The following sections will describe each module that makes up the transformer model in detail.

### 2.1 Attention modules

**Fig. 2.**
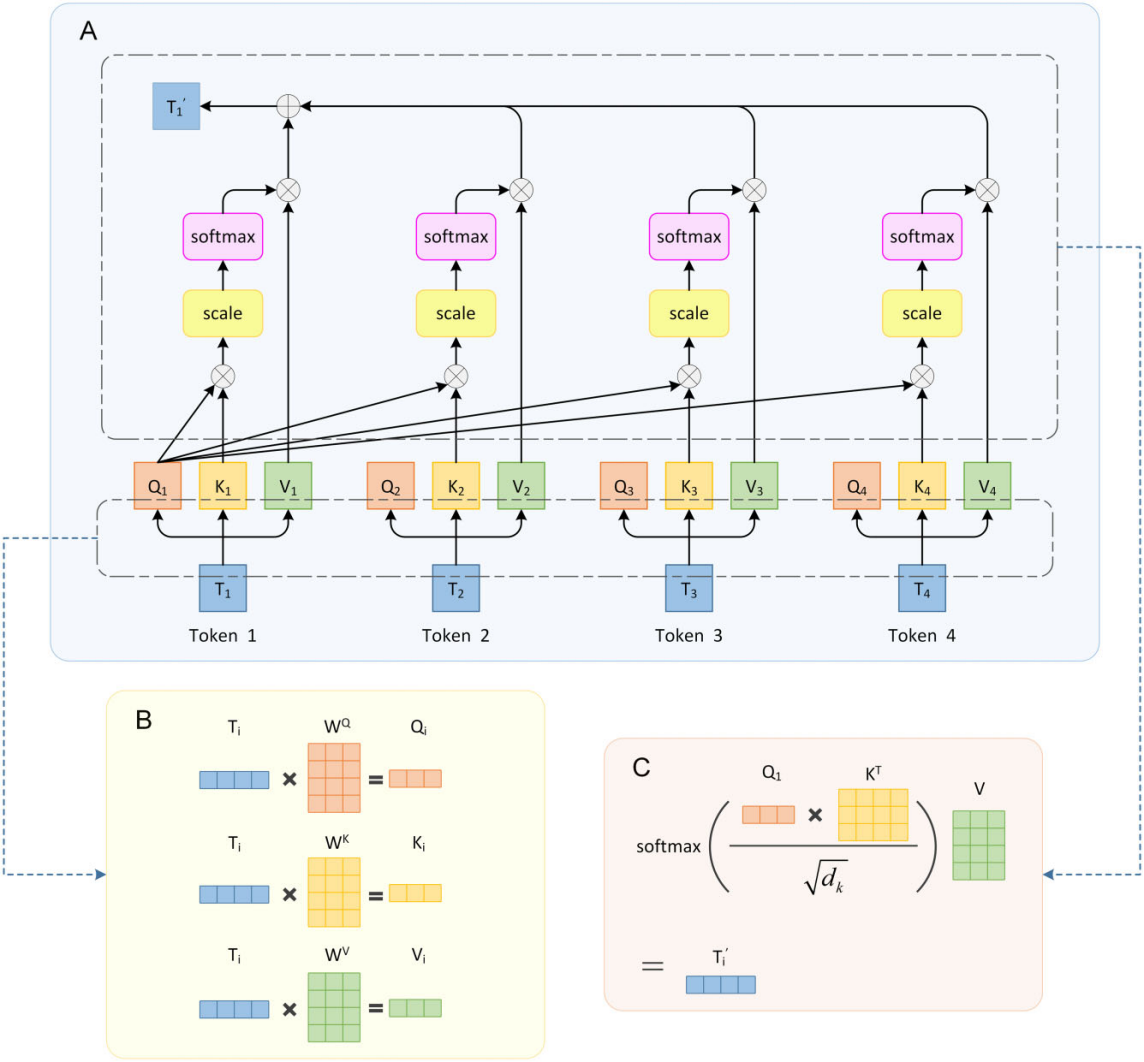
The example illustration of calculating self-attention. (**A**) The process of computing the output embedding of token $T_{1}$ in a single attention head. $T_{i(i=1,2,3,4)}$ represents the embeddings corresponding to the *i*th token in the input sequence. $T_{1}^{\prime }$ is the output corresponding to $T_{1}$*.* Each embedding in the input sequence needs to be multiplied with the three parameter matrices ${\;W}^{Q},\;W^{K}$ and $W^{V},$ respectively to obtain the corresponding query vector, key vector and value vector. (**B**) The figure complements the process of generating the *i*th (*i* = 1,2,3,4) token’s corresponding query vector $Q_{i}$, key vector $K_{i}$ and value vector $V_{i}$. Each attention head has its own set of three learnable parameter matrices ${\;W}^{Q},\;W^{K}$ and $W^{V}$. (**C**) If the key vectors of all tokens are concatenated into a matrix K by row and all value vectors are concatenated into a matrix V by row, the process of calculating $T_{1}^{\prime }$ in part A can be expressed as the formula in part C using matrix operations, where $K^{T}$ is the transpose of K and $d_{k}$ is the dimension of the key vector

The key innovation in transformer is the multi-head self-attention layer, which can relate all relevant tokens to better encode every word in the input sequence (Lin *et al.*, 2017). The self-attention layer takes a sequence of tokens as input (tokens equivalent to words in the language) and learns sequence-wide context information. Multi-head represents multiple simultaneous attention heads. Figure 2 shows the example process of a single attention head in calculating the first token $T_{1}’$s output embedding in a sequence composed of four tokens.

Before calculating the attention function, each token embedding will be transformed into the corresponding query vector, the key vector of dimension${\;d}_{k}\;$and the value vector of the dimension$\;d_{v}\;$by multiplying with three randomly initialized learnable parameter matrices $W^{Q},\;W^{K}\;\mathrm{and}\;W^{V}$. Then, the attention head will compute the dot products of the query with all keys and divide each by$\;\sqrt{d_{k}}\;$and apply a softmax function to obtain the weights on these values (Vaswani *et al.*, 2017). Through this process, the attention function can be described as mapping a query vector and a set of key-value pairs to an output vector that contains information for the entire sequence. As is seen in Figure 2, the output of the attention function is the weighted sum of these values. The weight assigned to each value is computed by a compatibility function of the query with the corresponding key (Vaswani *et al.*, 2017).

In the parallel computation of the attention function, a set of query vectors is packed into a matrix Q. These key and value vectors are also packed together into matrices K and V. In practice, the attention function is computed as follow:

(1) $$\mathrm{Attention}\left(Q,\;K,\;V\right)=\mathrm{softmax}\left(\frac{QK^{T}}{\sqrt{d_{k}}}\right)V.$$

When being generalized to multi-head attention with h heads, the results of multiple heads assigned different parameters${\;W}^{Q},\;W^{K}\;\mathrm{and}\;W^{V}$ are concatenated, and once again projected with parameter, resulting in the final values, as depicted as follows:

(2) $$\begin{array}{c} \mathrm{MultiHead}\left(Q,\;K,\;V\right)=\mathrm{Concat}({\mathrm{head}}_{1},\ldots {,\mathrm{head}}_{h})W^{O}\\ \mathrm{where}\;{\mathrm{head}}_{i}=\mathrm{Attention}\left(QW_{i}^{Q},\;KW_{i}^{K},\;VW_{i}^{V}\right) \end{array}.$$

### 2.2 Position-wise feed-forward networks

Except for the attention sub-layer, each block of the encoder and decoder contains a fully connected feed-forward network (FFN) (Skansi, 2018), which is applied identically to each token. This layer consists of two linear transformations with rectified linear unit (ReLU) activation in the middle (Vaswani *et al.*, 2017), where $\;W_{1}$,$\;b_{1}$,$\;W_{2}\;$and$\;b_{2}\;$are learnable parameters.

(3) $$\mathrm{FFN}\left(x\right)=\max\left(0,\;xW_{1}+b_{1}\right)W_{2}+b_{2}.$$

### 2.3 Residual connection and layer normalization

Each encoder contains two residual connection and layer normalization layers, and they are applied on both multi-head self-attention and FFN. The calculation formulas are as follows:

(4) $$\mathrm{LayerNorm}\left(X+\mathrm{MutiHeadAttention}\left(X\right)\right),$$

(5) $$\mathrm{LayerNorm}\left(X+\mathrm{FeedForward}\left(X\right)\right).$$

X represents the input of multi-head self-attention or FFN, which is added to the output and forms a residual connection. For the deep network, the residual connection can help fend against vanishing and exploding gradients by keeping the original input (Zhang *et al.*, 2018). Layer normalization can accelerate the training process of the model by normalizing the output of the former layers to make it converge faster (Ba *et al.*, 2016).

### 2.4 Position encodings

Since transformer uses pure self-attention without recurrence or convolution to capture connections between tokens, it cannot identify the order of the tokens in the sequence. Therefore, transformer adds position encodings to the input embeddings (Liu *et al.*, 2020) to reflect the absolute or relative position of the tokens in the sequence. The absolute position encoding informs the transformer architecture of the absolute position of each token in the input sequence, while the relative position encoding acts as a self-attention mechanism, informing the transformer architecture of the distance between two tokens (Ke *et al.*, 2021). The input for the first transformer encoder layer is the sum of the input embedding and the position encoding.

### 2.5 Encoder and decoder

Using the components above, the encoder encodes the input sequence and passes the output intermediate sequence to the decoder, and the decoder decodes the intermediate sequence and outputs the sequence we need. The encoder consists of several identical blocks consisting of one attention sub-layer and a feed-forward layer (Fig. 1C). The decoder inserts one more attention sub-layer between the original two sub-layers to perform multi-head attention over the output of the encoder stack (Fig. 1C).

**Fig. 3.**
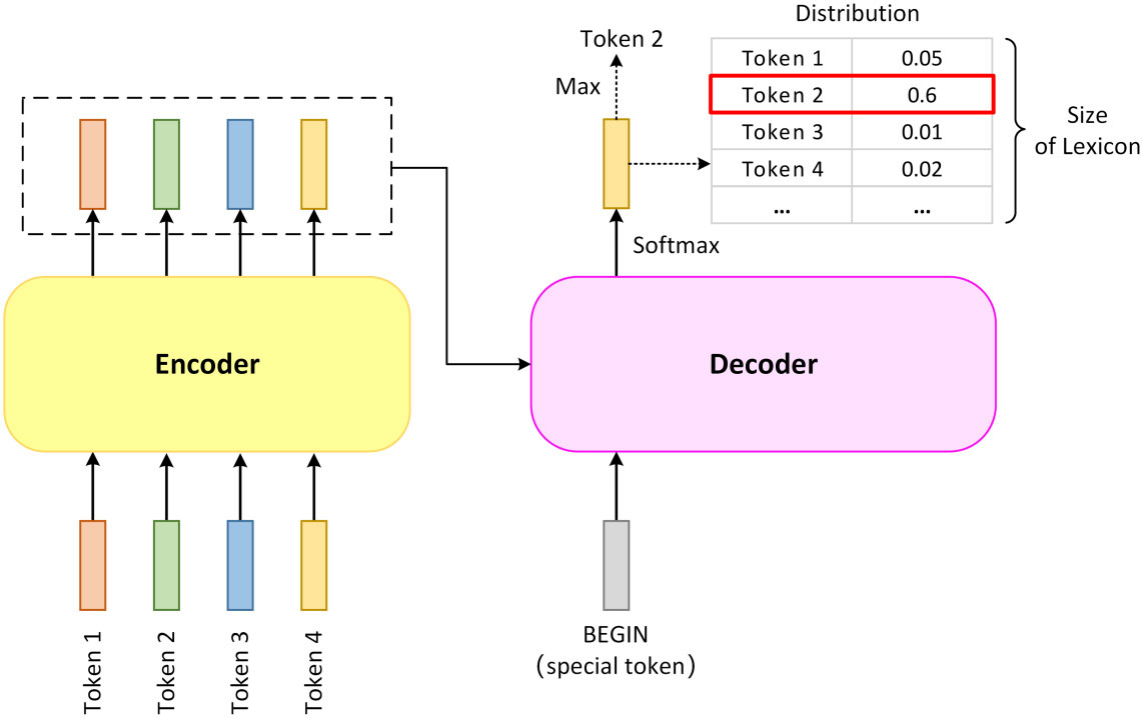
The first step of the decoding process. The decoder predicts which token to output with its input and the output of the encoder. The decoder takes a special token ‘BEGIN’ as input, combining it with the encoder’s output to generate the probability distribution vector. The length of this vector is the size of the lexicon, and each dimension of the output probability distribution vector represents the probability of a certain token in the lexicon. The output vector is then applied to a softmax function to generate a probability distribution, and the token in the lexicon with the highest probability is the corresponding output, which is also the first token in the final output sequence

Decoding the intermediate output of the encoder into a new sequence can be considered as a translation process. First, the decoder takes a special token ‘BEGIN’ as input, combining it with the encoder’s output sequence to produce a vector after passing through the inner blocks of the decoder and a linear layer. The length of this vector is the size of the lexicon. Then, a softmax function is applied to the output vector to generate a probability distribution, and the token in the lexicon with the highest probability is the output, which is also the first token in the final output sequence (Fig. 3).

**Fig. 4.**
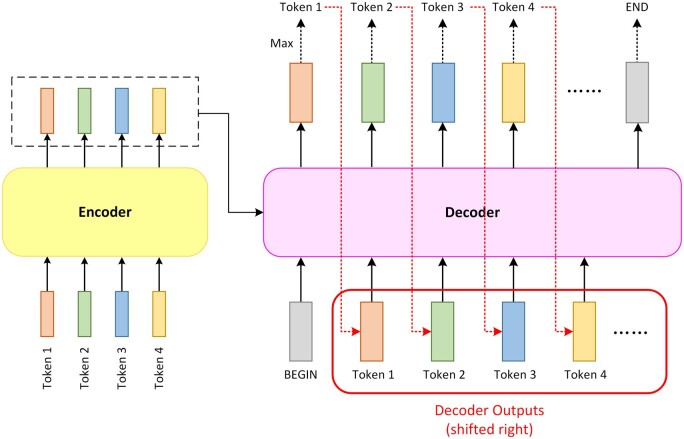
The process of decoder looping to produce the final output one by one, also known as the autoregressive process. In the same way as the first decoding round shown in Figure 3, each round decoder will generate a predicted probability distribution vector. Predicted tokens generated by ‘BEGIN’ in the first round will be appended to the sequence containing the ‘BEGIN’ token as the second round of the decoding process’s input. This process will be repeated, adding the new output into the input sequence. Because of the extra ‘BEGIN’ token, the decoder’s input is shifted one position to the right. In each decoding round, only the predicted tokens already decoded so far together with the special token ‘BEGIN’ are received as input to generate the new predicted token. It is worth mentioning these predicted tokens are not always correct, and a misprediction in the current round may affect the decoding correctness in the subsequent rounds. To end the loop, an ‘END’ token is added to the lexicon. When the output token is ‘END’ the loop stops, resulting in the complete final output sequence

This output token will be appended to the sequence containing the ‘BEGIN’ token as the next round of the decoding process’s input. This process will be repeated, appending the new output into the input sequence. To end the loop, an ‘END’ token is appended to the lexicon. The loop stops when the output token is ‘END’, resulting in the complete final output sequence. Because of the extra ‘BEGIN’ token, the decoder’s input is shifted one position to the right (Fig. 4).

**Fig. 5.**
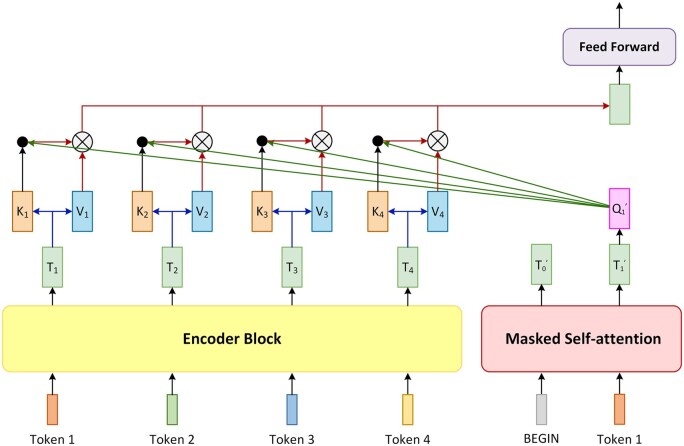
Structure of the cross-attention layer. The encoder block in this figure refers to a certain block in encoder whose output participate in cross-attention with the decoder. Masked self-attention refers to the first attention sub-layer in decoder block. $T_{i(i=1,2,3,4)}$ is the *i*th token’s output of the encoder block shown in this figure and also the *i*th token’s input of next encoder block. $K_{i(i=1,2,3,4)}$ and $V_{i(i=1,2,3,4)}$ are the key matrix and the value matrix of $T_{i}$. $Q_{1}^{'}$ is the corresponding query matrix of $T_{1}^{'}$, which is the first token’s output of masked self-attention. Cross-attention uses the decoder’s query and the encoder’s keys and values to calculate the attention function, and the output of cross-attention will be fed into the feed-forward layer in decoder block

It is worth mentioning that when generating an output token, the input sequence only contains the tokens before it. When passing through the first attention layer, the queries, values and keys after this token will be masked and will not participate in the attention calculation. The decoder’s input in the current round, which is the input of the previous round appending the output of the previous round, generates the vector of the corresponding position after passing through the masked self-attention layer. This vector will be multiplied by a transition matrix to obtain the query matrix of the second attention layer, which is also called the ‘cross-attention layer’ (Fig. 5).

In the cross-attention layer, the key matrices and value matrices in the attention function are provided by the output sequence of the encoder, while the query matrix is transformed from the output of the masked attention layer. Calculating cross-attention is the same as self-attention, except that the source of the query matrix is different. The output of the cross-attention layer also goes through a feed-forward layer. After that, it will be fed into the last linear layer and the softmax function to produce the final output of the round.

## 3 Bioinformatics applications of transformer-based language models

**Fig. 6.**
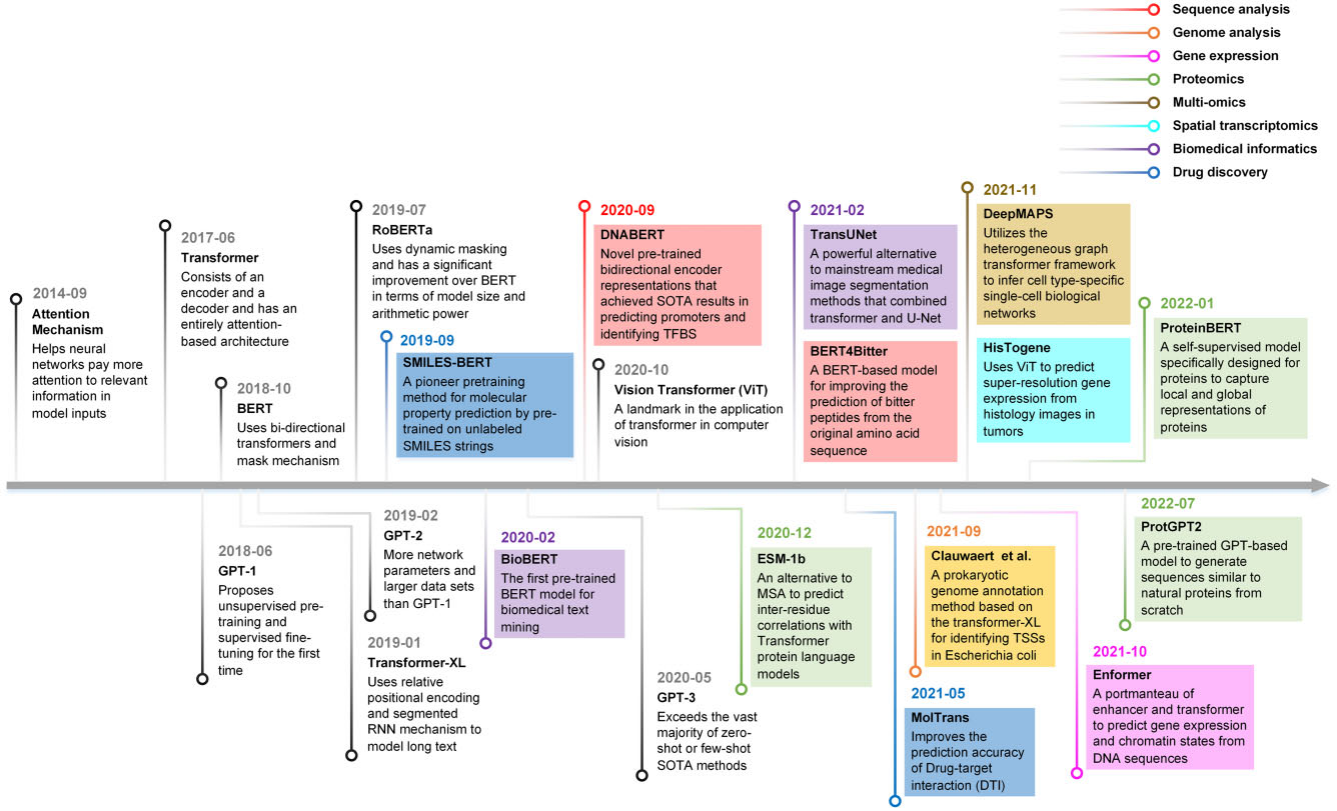
An overview of important works related to TRANSFORMER. Different bioinformatics application models are represented chronologically by different colored lines. Following the prominent progress in the past years, Transformer in bioinformatics will embrace great advancement in the upcoming years. SOTA, state-of-the-art

**Fig. 7.**
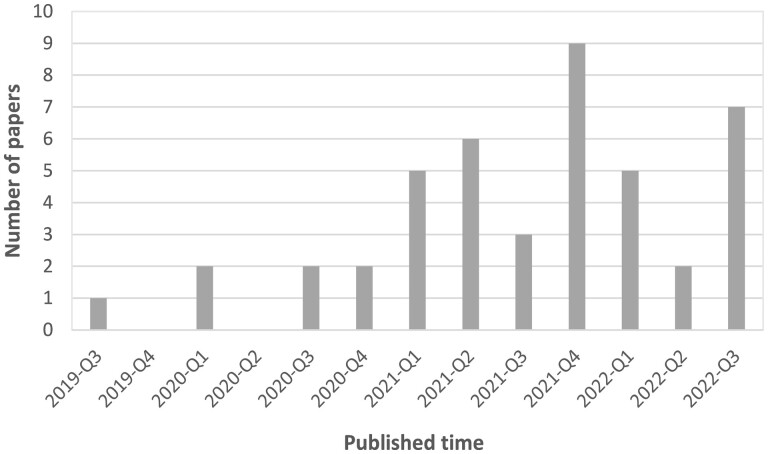
Distribution of selected papers published in recent years. Most papers (84.1%) were published after 2021, with the highest number of publications registered in 2021 (23 papers). Qx, x^th^ quarter of the year

This section summarizes and compares representative works in different fields of bioinformatics applications (Table 1), lists important works related to transformer (Fig. 6) and identifies their main focuses and benefits, e.g. improving model accuracy, reproducibility and interpretability. The number of transformer-based applications over the past 3 years (Fig. 7) suggests a growing interest in the field of bioinformatics.

**Table 1.** Summary and comparison of the representative applications of transformer-based language models in different fields of bioinformatics

Field	Paper	Pre-trained model? (Y/N)	Main focus	Data repositories address
Sequence analysis	Ji *et al.* (2021)	Y	Novel pre-trained bi-directional encoder representations that achieved state-of-the-art results in predicting promoters and identifying TFBSs	https://github.com/jerryj1993/DNABERT
	Lee *et al.* (2021)	Y	A transformer architecture based on BERT and 2D CNN to identify DNA enhancers from sequence information	https://github.com/khanhlee/bert-enhancer
	Zhang *et al.* (2021b)	Y	A transformer architecture based on BERT and stacking ensemble to identify RNA N7-Methylguanosine sites from sequence information	NA
	Charoenkwan *et al.* (2021)	Y	A bi-directional encoder representation from BERT-based model for improving the prediction of bitter peptides from the original amino acid sequence	http://pmlab.pythonanywhere.com/BERT4Bitter
	Qiao *et al.* (2022)	Y	Prediction of lysine crotonylation sites by a transfer learning method with pre-trained BERT models	http://zhulab.org.cn/BERT-Kcr_models/
Genome analysis	Clauwaert *et al.* (2021)	N	A prokaryotic genome annotation method based on the transformer-XL neural network framework for identifying TSSs in Escherichia coli	https://github.com/jdcla/DNA-transformer
	Raad *et al.* (2022)	N	A full end-to-end deep model based on transformer for prediction of pre-miRNAs in genome-wide data	https://github.com/sinc-lab/miRe2e
	Chen *et al.* (2022b)	N	Prediction of EPI in different cell types by capturing large genome contexts	https://github.com/biomed-AI/TransEPI
	Baid *et al.* (2022)	N	A gap-aware transformer–encoder for sequence correction trained by an alignment-based loss	https://github.com/google/deepconsensus
	Mo *et al.* (2021)	Y	Prediction of interactions between regulatory elements by pre-training large-scale genomic data in a multi-modal and a self-supervised manner	NA
Gene expression	Avsec *et al.* (2021)	N	A portmanteau of enhancer and transformer to predict gene expression and chromatin states from DNA sequences	https://github.com/deepmind/deepmind-research/tree/master/enformer
	Khan and Lee (2021)	N	Transformer for the gene expression-based classification of lung cancer subtypes that solved the complexity of high-dimensional gene expression through a multi-headed self-attention module	NA
	Yang *et al.* (2022)	Y	Single-cell bi-directional encoder representations from transformers for cell type annotation, new cell type discovery, handling of batch effects, and improving model interpretability.	https://github.com/TencentAILabHealthcare/scBERT
Proteomics	Cao and Shen (2021)	N	A high-throughput Transformer-based protein function annotator with both accuracy and generalizability	https://github.com/Shen-Lab/TALE
	Rao *et al.* (2020)	Y	An alternative to MSA to predict inter-residue correlations in an end-to-end manner with Transformer protein language models	https://github.com/facebookresearch/esm
	Rives *et al.* (2021)	Y	Learning protein biological structure and function from UniRef dataset using pre-trained Transformer	https://github.com/facebookresearch/esm
	Zhang *et al.* (2021a)	N	Jointly considering information of all homologous sequences in MSA to capture global co-evolutionary patterns	https://github.com/microsoft/ProteinFolding/tree/main/coevolution_transformer
	Elnaggar *et al.* (2022)	Y	Understanding the language of life with transformer-based protein language models through self-supervised learning	https://github.com/agemagician/ProtTrans
	Brandes *et al.* (2022)	Y	A self-supervised deep language model specifically designed for proteins to capture local and global representations of proteins in a natural way	https://github.com/nadavbra/protein_bert
	Park *et al.* (2022)	Y	A sequence-based pre-trained BERT model improved linear and structural epitope prediction by learning long-distance protein interactions effectively	NA
	Ferruz *et al.* (2022)	Y	A pre-trained GPT-based model to generate sequences similar to natural proteins from scratch	https://huggingface.co/docs/transformers/main_classes/trainer
	Castro *et al.* (2022)	N	An autoencoder based on transformer with a highly structured latent space trained to jointly generate sequences and predict fitness	https://github.com/KrishnaswamyLab/ReLSO-Guided-Generative-Protein-Design-using-Regularized-Transformers
Multi-omics	Tao *et al.* (2020)	Y	Prediction of multiple cancer phenotypes based on somatic genomic alterations via the genomic impact transformer	https://github.com/yifengtao/genome-transformer
	Jurenaite *et al.* (2022)	Y	Applying Transformer-based deep neural network on mutomes and transcriptome counting for tumor type classification	https://github.com/danilexn/nebis
	Kaczmarek *et al.* (2021)	N	The use of graph transformer network (GTN) for cancer classification and interpretation	NA
	Ma *et al.* (2021)	N	Utilizing the heterogeneous graph transformer framework to infer cell type-specific single-cell biological networks	https://github.com/OSU-BMBL/deepmaps
Spatial transcriptomics	Pang *et al.* (2021)	N	Usage of Vision Transformer (ViT) to predict super-resolution gene expression from histology images in tumors	https://github.com/maxpmx/HisToGene
	Zeng *et al.* (2022)	N	Spatial transcriptomics prediction from histology jointly through transformer and graph neural networks	https://github.com/biomed-AI/Hist2ST
Biomedical informatics	Lee *et al.* (2020)	Y	The first pre-trained biomedical language representation model for biomedical text mining	https://github.com/dmis-lab/biobert
	Rasmy *et al.* (2021)	Y	Pre-training contextualized embeddings on large-scale structured electronic health records for disease prediction that used the International Classification of Diseases (ICD) codes	https://github.com/ZhiGroup/Med-BERT
	Wang *et al.* (2021)	Y	An innovative AlBERT-based causal inference model of clinical events	https://github.com/XingqiaoWang/DeepCausalPV-master
	Chen *et al.* (2021a)	Y	A powerful alternative to mainstream medical image segmentation methods that combined transformer and U-Net	https://github.com/Beckschen/TransUNet
	Chen *et al.* (2021b)	Y	Using ViT for the first time in self-supervised volumetric medical image registration	https://bit.ly/3bWDynR
Drug discovery	Wang *et al.* (2019)	Y	A pioneer pre-training method for molecular property prediction by pre-trained on unlabeled SMILES strings	https://github.com/uta-smile/SMILES-BERT
	Rong *et al.* (2020)	Y	A new GNN/Transformer architecture that learned rich molecular structure and semantic information from large amounts of unlabeled data	https://github.com/tencent-ailab/grover
	Chithrananda *et al.* (2020)	Y	Utilizing RoBERTa-based transformer for molecular property prediction	https://huggingface.co/seyonec
	Wu *et al.* (2022)	Y	Presenting new pre-training strategies that allowed the model to extract molecular features directly from SMILES	https://github.com/wzxxxx/Knowledge-based-BERT
	Li *et al.* (2022)	Y	A novel knowledge-guided pre-training framework of graph transformer for molecular property prediction	https://github.com/lihan97/KPGT
	Huang *et al.* (2021)	N	Improving the prediction accuracy of DTI by knowledge-inspired representation, interaction modeling modules and an augmented transformer encoder	https://github.com/kexinhuang12345/moltrans
	Kalakoti *et al.* (2022)	N	A modular framework that employing transformer-based language models for DTI prediction	https://github.com/TeamSundar/transDTI
	Jiang *et al.* (2022)	N	An end-to-end deep transformer-based learning model that used cancer cell transcriptome information and chemical substructures of drugs to predict drug response	https://github.com/jianglikun/DeepTTC
	Bagal *et al.* (2022)	Y	A small version of the GPT model for molecular generation	https://github.com/devalab/molgpt
	Grechishnikova (2021)	N	A *de novo* drug generation model based on transformer architecture	https://github.com/dariagrechishnikova/molecule_structure_generation

*Note*: The papers are sorted by their appearance in this review and divided into different categories based on their research field.

### 3.1 Sequence analysis

Biological sequence analysis, including DNA, RNA and protein sequence analysis, represents one of the fundamental applications of computational methods in molecular biology. Traditional sequence analysis methods rely heavily on k-mers frequency (Koonin and Galperin, 2003b), which is not able to capture distant semantic relationships of gene regulatory code. Deep learning models like CNN also have problems capturing semantic dependency within long-range contexts (Tang *et al.*, 2018), as their capability to extract local features is limited by the filter size. The RNN-based models (e.g. LSTM and GRU) are developed to capture long-range dependency; however, it is difficult for them to perform large-scale learning due to their limited degree of parallelization. In addition, existing models generally require large amounts of labeled data, which is difficult to obtain in bioinformatics research (Butte, 2001).

**Fig. 8.**
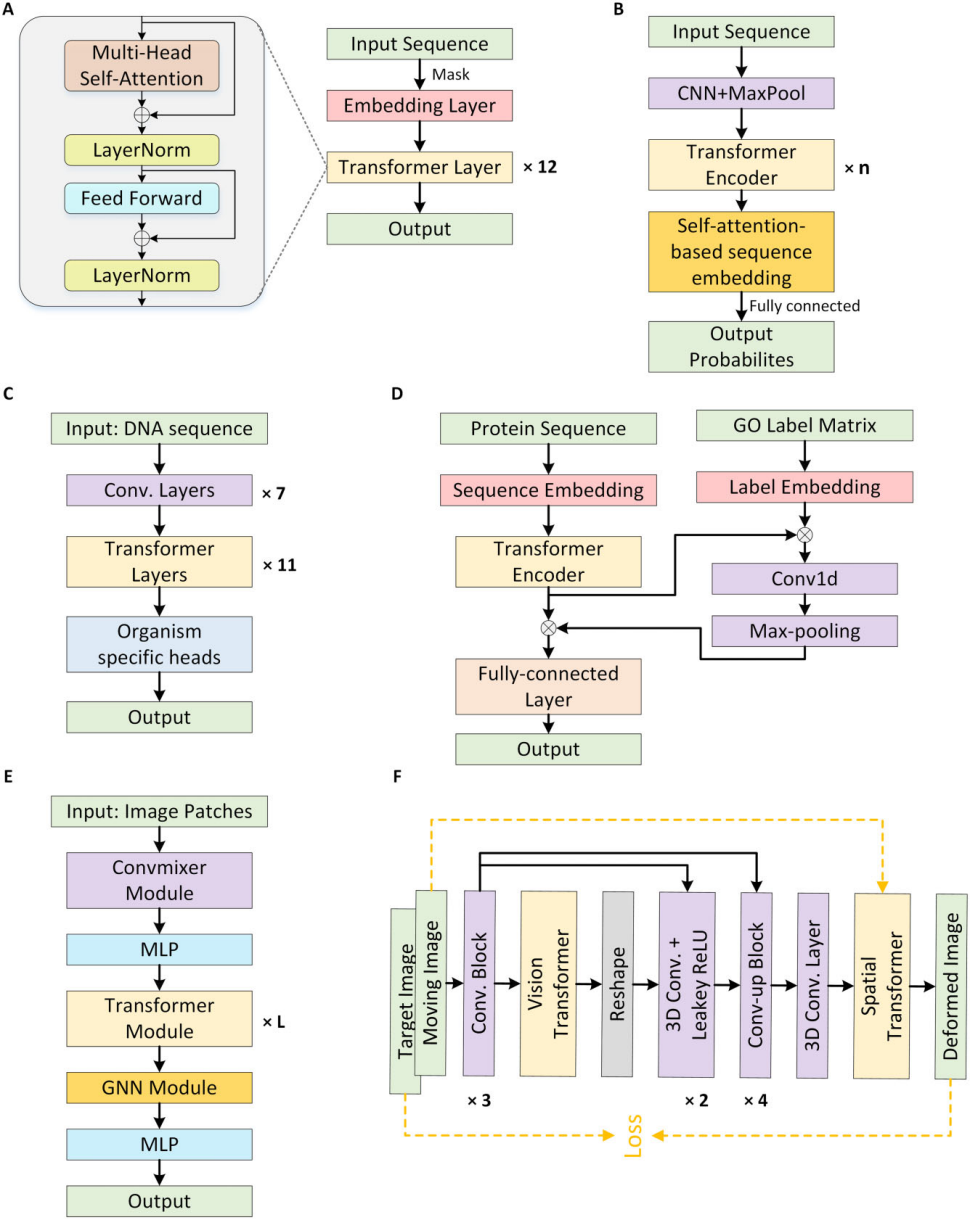
Several typical models of Transformer applied to bioinformatics including the frameworks of (**A**) DNABERT, (**B**) TransEPI, (**C**) Enformer, (**D**) TALE, (**E**) Hist2ST and (**F**) ViT-V-Net

Considering the large amount of unlabeled genomic sequences, transformer-based pre-trained language models are well-suited for DNA sequence analysis and have received increasing attention for their significant improvement over other traditional or deep learning models. DNABERT (Ji *et al.*, 2021), a novel pre-trained bi-directional coding representation, used tokenized k-mer sequences as input for the BERT model (Fig. 8A). DNABERT utilized context information in DNA sequences and achieved state-of-the-art results in downstream tasks such as predicting promoters and identifying transcription factor binding sites (TFBSs). Another example is to use the multi-language model based on BERT by converting DNA sequences into a numerical matrix of constant size for the prediction of enhancers (Lee *et al.*, 2021). Compared with the most advanced features in bioinformatics, BERT-based features increased the sensitivity, specificity, accuracy and Matthews correlation coefficient (MCC) by 5–10%.

Compared with DNA sequences, RNA sequences provide additional transcription information. While traditional methods still rely on manually curated RNA sequence features, deep learning models enable automatic feature extraction (Urda *et al.*, 2017). BERT-m7G was a transformer model based on BERT and used a stacking ensemble to identify RNA N7-methylguanosine (m7G) sites from RNA sequence information (Zhang *et al.*, 2021b). N7-methylguanosine is one of the most prevalent RNA post-transcriptional modifications and plays an important role in the regulation of gene expression. The experimental results showed that the identification performance of BERT-m7G obviously exceeded the existing prediction methods, with the accuracy increasing by 3–20.7% and the MCC improving by 0.06–0.415.

Protein sequence analysis can be regarded as an extension of DNA sequence analysis (von Heijne, 1992), but it is much more complicated than DNA sequence analysis because polymers are composed of 20 amino acids (Karlin and Ghandour, 1985). The analysis of protein sequences can better capture the relationships between protein sequences and the spatial structure of proteins and provide a theoretical basis for further study on protein function and structure (Findlay *et al.*, 1995; Ponting and Birney, 2005). For example, bitter peptides are oligopeptides with a bitter taste usually produced during food fermentation and protein hydrolysis (Karametsi *et al.*, 2014), which are useful for drug development since diluting the bitterness of drugs can increase patients’ willingness to take medicine. BERT4Bitter was proposed to predict bitter peptides directly from the original amino acid sequence without using any structural information (Charoenkwan *et al.*, 2021). It was the first study to identify bitter peptides using the NLP-inspired model and feature encoding. In another study, Qiao *et al.* (2022) established a more effective predictor for protein lysine crotonylation sites (Kcr), which is one of the most important post-translational modifications, by pre-training BERT model. The authors converted each amino acid into a word as the input to the pre-trained BERT model. The features encoded by BERT were extracted and then fed to the BiLSTM network (Zeng *et al.*, 2016) to construct the final model.

### 3.2 Genome analysis

Although sequence analysis contributes significantly to biological discovery, genome analysis is also essential to capture the full repertoire of information encoded in the genome (Koonin and Galperin, 2003a). Genome analysis explains the appearance of tumors or phenotypes from the DNA level, including gene mutations, deletions, amplifications (Feuk *et al.*, 2006) and epigenetic modifications (e.g. DNA methylation) (Nikpay *et al.*, 2015; Portela and Esteller, 2010).

Several scratch-trained methods based on the Transformer model have been developed to this end. For example, Clauwaert *et al.* (2021) proposed a prokaryotic genome annotation method based on the Transformer-XL neural network framework, which was designed to identify transcription start sites (TSSs) for the transcription process in *Escherichia coli*. Beyond the application to genome annotation, some studies also applied Transformers to the prediction of small-RNA sequences. For example, MiRe2e, a full transformers-based end-to-end deep model, was developed to predict pre-miRNAs (Raad *et al.*, 2022). MiRe2e showed its advantages in two aspects: (i) It can receive raw genome-wide data without any preprocessing or secondary structure prediction; (ii) It identified all pre-miRNA sequences in the genome with high accuracy and recall. In another study, TransEPI (Chen *et al.*, 2022b) was developed based on enhancer–promoter interaction (EPI) datasets derived from Hi-C or ChIA-PET data to predict EPI in different cell types by capturing large genome contexts (Fig. 8B). This model not only achieved state-of-the-art results on experimental datasets [the area under the precision-recall curve (auPRC) of TransEPI increased by an average of 28.1% compared to the second-best baseline] but has also been extended to the interpretation of disease-related non-coding mutations. Last but not least, Google’s Andrew Carroll research group recently developed DeepConsensus, which uses the alignment-based loss to train gap-aware transformer-encoders for sequence correction (Baid *et al.*, 2022). Compared to methods using pbccs (standard approach to consensus generation), DeepConsensus reduced errors in reads (small genome fragments from sequencing sampling) by 41.9%, and improved the adjacency, completeness and correctness of genome assembly.

In addition, the Transformer-based pre-trained models were also used to predict the interactions between regulatory elements. One example is GeneBERT (Mo *et al.*, 2021). It was proposed to address the problem that traditional methods rarely consider the interactions among multiple regulatory elements in the regulatory genome. GeneBERT was pre-trained using large-scale genomic data in a multi-modal and self-supervised manner, in which three pre-training tasks: sequence pre-training, region pre-training and sequence-region matching, were proposed to improve the robustness and generalization ability of the model.

### 3.3 Gene expression

Gene expression data (Brazma and Vilo, 2000), like RNA-sequencing (RNA-seq) and single-cell RNA-seq (scRNA-seq) (Kolodziejczyk *et al.*, 2015; Ozsolak and Milos, 2011), has been extensively studied to better understand complex diseases and to identify biomarkers that can guide therapeutic decision-making (Goeman and Bühlmann, 2007). They have substantial applications in clinical medical diagnosis, drug efficacy judgment and revealing the mechanism of disease (Rotter *et al.*, 2010; Rung and Brazma, 2013).

To examine how non-coding DNA determines gene expression in different cell types, DeepMind proposed a noteworthy model Enformer (Avsec *et al.*, 2021). Due to the limitations of previous convolutional operations in modeling the effects of distal enhancers and insulators on gene expression, Enformer introduced the transformer structure (Fig. 8C), greatly increasing the receptive field of the network (from 20 to 100 kb). Enformer not only greatly improved the accuracy of predicting gene expression from DNA sequences, with the mean correlation increasing from 0.81 to 0.85, but also represented an important step forward in human understanding of the complexity of genome sequences. Furthermore, Enformer predicted EPI directly from DNA sequences by leveraging the self-attention mechanism and provided a more accurate prediction of mutation effects through direct mutation analysis and population eQTL studies (Liu *et al.*, 2022).

In addition to predicting the effect of non-coding DNA on gene expression, transformer-based models have been widely used to predict cancer subtypes according to gene expression data. Gene transformer used the multi-headed self-attention module to solve the complexity of high-dimensional gene expression for joint classification of lung cancer subtypes (Khan and Lee, 2021). Compared with traditional classification algorithms, the proposed model achieved an overall performance improvement in all evaluation metrics, with 100% accuracy and zero false-negative rates on most datasets.

scRNA-seq is a revolutionary technology in the life science field. One of the latest studies innovatively proposed scBERT model for single-cell annotation (Yang *et al.*, 2022). It was the first time to apply Transformer in scRNA-seq data analysis. Following BERT’s pre-training and fine-tuning paradigm, scBERT reused large-scale unlabeled scRNA-seq data to accurately capture the expression information of a single gene and the gene–gene interactions and revealed single-cell type annotation with high interpretability, generalization and stability.

### 3.4 Proteomics

The essential task of proteomics is to understand protein dynamics in complex systems and diseases (Larance and Lamond, 2015; Rix and Superti-Furga, 2009). Protein sequences can be viewed as a concatenation of letters from the amino acids, analogously to human languages. These letters form secondary structural elements (‘words’), which assemble to form domains (‘sentences’) that undertake a function (‘meaning’) (Ofer *et al.*, 2021). With the extraordinary advances in the NLP field in understanding and generating language with human-like capabilities, some language models open a new door to figuring out protein-related problems from sequences alone, such as protein sequence representation, post-translational modifications, protein function annotation and protein design.

Especially, transformer has served as a key technique for addressing various aspects of proteomics data analysis. The work of Cao and Shen (2021) exemplified the application of transformer to protein function annotation, a critical step in identifying the overall functional distribution of differentially expressed proteins. Specifically, the model obtained embedding by using sequence inputs, hierarchical function labels and their joint similarity to measure the contribution of each amino acid to each label. The final model was shown to be a high-throughput protein function annotator with high accuracy and generalizability (Fig. 8D).

The measurement of amino acid proximity of proteins is called the inter-residue contact map, which well characterizes the structural information of proteins. Most of the top-performing models for protein contact prediction use multiple sequence alignment (MSA), which improves protein 3D structure prediction by analyzing residue co-evolution information in sequences. Facebook AI Research proposed ESM-1b (Rao *et al.*, 2020), a method alternative to MSA using the transformer to predict inter-residue correlations in an unsupervised manner. Subsequently, they applied ESM-1b to the UniRef dataset (250M protein sequence) for biochemical properties analysis, secondary and tertiary structure prediction and mutation analysis to fully explore the rich information contained in protein sequences (Rives *et al.*, 2021). Since the prevalence of non-homologous residues and gaps in MSA may lead to erroneous estimation of residue co-evolution, Co-evolution Transformer (CoT) was proposed to reduce the impact of non-homologous information (Zhang *et al.*, 2021a). CoT selectively aggregated features from different homologous sequences by assigning smaller weights to non-homologous sequences or residue pairs. By jointly considering the information of all homologous sequences in MSA, CoT was able to capture global co-evolutionary patterns.

There are some important works related to protein sequence embedding in recent years (Alley *et al.*, 2019; Elnaggar *et al.*, 2022; Heinzinger *et al.*, 2019; Unsal *et al.*, 2022). Elnaggar *et al.* (2022) proposed to make transformer-based protein language models capture constraints relevant for protein structure and function by transfer learning (using trained embeddings as input to subsequent supervised training). The researchers trained two auto-regressive models (Transformer-XL and XLNet) and four auto-encoder models (BERT, ALBERT, ELECTRA and T5) on large-scale protein sequences and tested both residue-level (3-state accuracy Q3 = 81–87%) and protein-level (10-state accuracy: Q10 = 81%, 2-state accuracy Q2 = 91%) prediction tasks using the embeddings obtained from the language models above, and found that ProtT5 fine-tuned on UniRef50 without MSA outperformed ESM-1b and achieved the best performance.

Other transformer-based pre-trained models have also been widely used in proteomics research. ProteinBERT is a model specifically designed for proteins (Brandes *et al.*, 2022). The pre-training scheme combined language modeling with gene ontology (GO) (Ashburner *et al.*, 2000; Stevens, 2000) annotation prediction. ProteinBERT aimed to capture local and global representations of proteins in a natural way, which allowed end-to-end processing of these types of input and output, making the model efficiently and flexibly adapt to long sequences. EpiBERTope (Park *et al.*, 2022) is a sequence-based pre-trained BERT model to predict both linear and structural epitopes. Epitopes are immunogenic regions of antigens that can be recognized by antibodies in a highly specific manner and trigger immune responses. EpiBERTope used a multi-headed attention mechanism to construct global dependencies for each amino acid in the protein sequences. In the fine-tuning stage, both linear and structural epitopes datasets were the input of EpiBERTope.

Beyond the applications mentioned above, transformer-based generative models began to be used for protein design in recent studies. Inspired by generative transformer-based language models (such as the GPT-X family), ProtGPT2 (Ferruz *et al.*, 2022) could generate sequences similar to natural proteins from scratch and thereby possesses the potential to solve many biomedical and environmental problems. Castro *et al.* (2022) proposed Regularized Latent Space Optimization (ReLSO), which combined the powerful encoding ability of the model with the capacity to generate low-dimensional latent representations with rich information. By simultaneously optimizing protein sequence generation and fitness landscape (Romero and Arnold, 2009) prediction, a latent space that contained rich information about sequence and fitness was explicitly created. In addition, the authors mentioned that ReLSO-like structures could be applied to other biomolecules such as DNA and RNA.

### 3.5 Multi-omics

The multi-omics analysis aims to better understand biological regulation by combining different types of omics data (Yang *et al.* 2019a). With the development of high-throughput sequencing technology, there is a growing interest in combining genomics with transcriptomics, proteomics and metabolomics together to understand the disease pathways and processes as a single type of omics data cannot capture the entire landscape of the complex biological networks (Castro-Vega *et al.*, 2015; Kang *et al.*, 2022).

The transformer-based model provides a new perspective for the analysis of various omics data in terms of diseases, while most conventional methods rarely take the relationships between different omics levels into account. To this end, Tao *et al.* (2020) proposed the genomic impact transformer (GIT). The GIT fine-tuned gene embeddings that were pre-trained by the ‘Gene2Vec’ algorithm in order to infer how somatic genomic alterations (SGAs) affect the function of cellular signaling systems and thus cause cancer by modeling the statistical relationship between SGAs events and tumor differentially expressed genes (DEGs). A recent article presented SetQuence and SetOmic (Jurenaite *et al.*, 2022), which applied transformer-based deep neural networks on mutome and transcriptome together, showing superior accuracy and robustness over previous baselines (including GIT) on tumor classification tasks.

Several applications in multi-omics made use of graph transformer networks (GTN) (Yun *et al.*, 2019). For instance, a novel method for cancer classification and interpretation (Kaczmarek *et al.*, 2021) could correctly model and interpret the interaction and biological communication between miRNAs and mRNAs to discover important miRNA-mRNA cancer pathways. Notably, although GTN was not superior to other baselines like GCN (Zitnik *et al.*, 2018), SVM (Cortes and Vapnik, 1995) and MLP (Kothari and Oh, 1993), it provided a high degree of interpretation of the results, as the attention of GTN could identify potential targeting pathways and biomarkers, which is almost impossible to be achieved by other models. DeepMAPS was a deep learning-based single-cell multi-omics data analysis platform that utilized the heterogeneous graph transformer framework to infer cell type-specific single-cell biological networks (Ma *et al.*, 2021). DeepMAPS can include all cells and genes in a heterogeneous graph to infer cell–cell, gene–gene and cell–gene relationships simultaneously.

### 3.6 Spatial transcriptomics

Spatially resolved transcriptomics has experienced significant progress in the biomedical research field with advances in imaging and next-generation sequencing technology (Reis-Filho, 2009). The relationship between cells and their relative positions in tissue samples is crucial for identifying intercellular communication networks and global transcriptional patterns, and understanding disease pathology. While single-cell transcriptome sequencing techniques address the issue of cell heterogeneity and allow us to identify cellular variants that play key roles in diseases (Faridani *et al.*, 2016), they cannot be targeted to specific spatial positions, resulting in the exploration of cell functions that are not yet particularly precise. Spatial transcriptomics not only provides information on the transcriptome data of the subject, but also locates its spatial location in the tissue, which is of great significance and thus provides a tremendous opportunity for many research fields such as oncology, neuroscience, immunology and developmental biology (Chen *et al.*, 2022a).

Transformer-based language models have been applied on this front to predict cell composition and gene expression in different areas of tissue. One example is HisTogene (Pang *et al.*, 2021), which employed Vision Transformer (ViT) (Dosovitskiy *et al.*, 2021), a state-of-the-art method for image recognition, to predict super-resolution gene expression from hematoxylin and eosin (H&E)-stained histology images. The model demonstrated favorable performance across datasets of 32 HER2+ breast cancer samples both in gene expression prediction and clustering tissue regions using the predicted expression. Based on this study, to capture 2D visual features of histology images and better highlight the explicit neighborhood relationships of image patches, the Hist2ST (Zeng *et al.*, 2022) model was developed for predicting RNA-seq expression from histology images (Fig. 8E). The model cropped histology images into patches at sequencing spots, learned 2D features in the image patches by convolutional operations and then captured global spatial dependencies between features using the transformer module while capturing explicit neighborhood relationships by graph neural networks (GNN) (Scarselli *et al.*, 2009). This study also proposed a self-distillation mechanism to mitigate the effects of small spatial transcriptomics data effectively.

### 3.7 Biomedical informatics

Biomedical informatics uses theories and techniques of computer science and other related disciplines’ research methods for developing innovative research and application in biomedical and clinical medicine (Boguski and McIntosh, 2003; Sarkar, 2010). The success of transformer-based language models has led researchers to focus on biomedical text and medical image processing, which again shows the superior performance of the Transformer.

One of the applications in biomedical text processing is BioBERT (Lee *et al.*, 2020), the first pre-trained BERT model for biomedical corpora. BioBERT initialized weights from general domain pre-trained BERT, trained on a large-scale biomedical corpus and fine-tuned on biomedical text mining tasks including NER (Marrero *et al.*, 2013), RE (Zhang *et al.*, 2017) and QA (Calijorne Soares and Parreiras, 2020). To enable deep learning models to predict disease status using limited training data, another study proposed Med-BERT (Rasmy *et al.*, 2021), a contextualized embedding model for pre-training on structured electronic health records (EHRs) data. In contrast to other medical pre-trained models that were trained on free text, this model was characterized by using the International Classification of Diseases (ICD) codes. After fine-tuning experiments on pancreatic cancer prediction and heart failure prediction in diabetic patients, Med-BERT was validated to be generalized on different sizes of fine-tuned training samples, which can better meet disease prediction research with small training datasets. Another promising application based on biomedical text data is an ALBERT-based model called InferBERT to predict clinical events and infer the causality (Wang *et al.*, 2021), which is a prerequisite for deployment in drug safety. As evaluated on two FDA Adverse Event Reporting System cases, the results showed that the number of causal factors identified by InferBERT for analgesics-related acute liver failure and Tramadol-related mortalities was 1.87 and 1.16 times higher than the second-best baseline, respectively.

Transformer has not only dominated the NLP field but has recently revolutionized the computer vision field (Han *et al.*, 2023; Khan *et al.*, 2022). Specifically, ViT applied Transformer to image classification tasks and achieved SOTA performance with less computational expense than other methods (Dosovitskiy *et al.*, 2021). Subsequent to this progress, TransUNet pioneered the pre-trained ViT for 2D medical image segmentation (Chen *et al.*, 2021a). It not only encoded image features as sequences to extract global context but also exploited low-level details for precise localization through a U-Net (Ronneberger *et al.*, 2015) hybrid network design. As a powerful alternative to mainstream medical image segmentation methods based on fully convolutional neural networks, TransUNet outperformed prior tools on tasks such as synapse multi-organ segmentation and cardiac segmentation, e.g. average dice score gained a range from 1.91% to 8.67%. ViT-V-Net (Chen *et al.*, 2021b) used ViT for the first time in self-supervised volumetric medical image (i.e. 3D images) registration (Fig. 8F). Combining the advantages of Transformer and V-Net (Milletari *et al.*, 2016), the network learned long-distance relationships between points in images while maintaining the flow of localization information.

### 3.8 Drug discovery

Despite progress in technology and enhanced knowledge of human disease, the translation of these advances into therapeutic benefits has been far slower than expected. The challenges facing the global pharmaceutical industry are multifold, including high attrition rates, increased time to bring new drugs to the market and changing regulatory requirements, which can all contribute to higher costs. A key issue in the early stage of drug design and discovery is the prediction of molecular properties and interactions (Lo *et al.*, 2018). While deep learning models have been widely applied to this end (Feinberg *et al.*, 2018; Liu *et al.*, 2019a; Wu *et al.*, 2018), the scarcity of labeled data remains a fundamental obstacle to accurate and efficient molecular property prediction. For this reason, large amounts of unlabeled data have been considered to improve the prediction performance on small-scale labeled data with the strength of transformer-based self-supervised pre-training.

Several momentous pre-training methods for molecular property prediction have been proposed, including SMILES-BERT (Wang *et al.*, 2019), GROVER (Rong *et al.*, 2020), ChemBERTa (Chithrananda *et al.*, 2020), K-BERT (Wu *et al.*, 2022) and KPGT (Li *et al.*, 2022). SMILES-BERT was pre-trained on large-scale unlabeled data by a Masked SMILES Recovery task by converting molecular formulas into SMILES strings (a kind of single-line text representation for the structure of molecular compounds) as input sequences (Wang *et al.*, 2019). The pre-trained model was fine-tuned with the labeled datasets and achieved excellent results on many datasets. However, SMILES-BERT lacks model interpretability since SMILES is not topology-aware and cannot explicitly encode the structural information of molecules. GROVER integrated Dynamic Message Passing Networks (Gilmer *et al.*, 2020) from GNNs and long-range residual connection into Transformer architecture to provide a more expressive molecular encoder and demonstrated clear improvement in molecular classification and regression tasks (Rong *et al.*, 2020). ChemBERTa utilized RoBERTa-based Transformer and evaluated the model with ROC–AUC metrics for MoleculeNet tasks (Chithrananda *et al.*, 2020). Although the experimental result was not state-of-the-art, ChemBERTa could scale the pre-training dataset well, with powerful downstream performance and practical attention-based visualization modality. K-BERT (Wu *et al.*, 2022) presented new pre-training strategies that allowed the model to extract molecular features directly from SMILES. The atomic feature prediction task enabled K-BERT to learn the initial atomic information that was extracted manually in graph-based approaches, the molecular feature prediction task enabled K-BERT to learn the molecular descriptor/fingerprint information that was extracted manually in descriptor-based approaches, and the contrastive learning task enabled K-BERT to better ‘understand’ SMILES through making the embeddings of different SMILES of the same molecule more similar. To alleviate the issues of the unclear definition of pre-training tasks and limited model capacity, Li *et al.* (2022) introduced KPGT, i.e. Knowledge-guided Pre-training of Graph Transformer for molecular graph representation learning and achieved state-of-the-art performance. KPGT proposed the Line Graph Transformer, which is a high-capacity model to emphasize the importance of chemical bonds and model the structural information of molecular graphs as line graphs. A knowledge-guided pre-training strategy based on generative self-supervised learning was then designed to exploit the molecular descriptors/fingerprints to guide the model to obtain plentiful structural and semantic information from large-scale unlabeled molecular graphs.

In addition to its role in molecular property prediction, transformer has been used in a wide range of applications to predict the interaction between biomolecules and compounds, e.g. drug–targeting interaction (DTI), which is a fundamental task for in silico drug discovery. Huang *et al.* (2021) proposed Molecular Interaction Transformer (MolTrans) to improve the accuracy of DTI prediction. With knowledge-inspired representation, interaction modeling modules and an augmented transformer encoder, MolTrans could extract semantic relationships between substructures from large amounts of unlabeled biomedical data. A recent study presented TransDTI (Kalakoti *et al.*, 2022), a modular framework that employs transformer-based language models to predict DTIs. TransDTI outperformed other descriptors and existing models including MolTrans. More recently, DeepTTA was released, which used cancer cell transcriptome information and chemical substructures of drugs to predict drug response (Jiang *et al.*, 2022). The model utilized transformers to mine drug features from substructures and a four-layer neural network to predict the transcriptomic data of anticancer drug response, making it easier to find effective cancer therapeutic drugs.

The generative models can produce molecules similar to but different from those in the training set by learning the distribution of the molecules in the training set. Another important development is that the transformer-based generative modeling brings new ideas to drug design. MolGPT is a small version of the GPT model for molecular generation (Bagal *et al.*, 2022). The model used masking self-attention mechanisms to make it easier to capture the long-range dependencies. In order to reduce the dependence on prior knowledge, such as the physical and chemical characteristics of proteins in the process of drug discovery, Grechishnikova (2021) proposed a *de novo* drug generation model based on transformer architecture. The goal of this model is to generate realistic lead compounds only using the amino acid sequence information of the target protein.

## 4 Challenges and opportunities

In this subsection, we discuss several key challenges and opportunities when applying transformer-based language models in bioinformatics research.

### 4.1 Heterogeneous training data

The rapid development of various types of omics technologies represented by high-throughput sequencing and mass spectrometry (Noor *et al.*, 2021) has made bioinformatics research obtain powerful data as input, with the result that the input of transformer in bioinformatics is not the same as it was originally applied in NLP. Instead, there is heterogeneous information, including text, code, graphs, etc. To fully capture the information in these heterogeneous data, both in-depth data preprocessing and model adaption may be needed. For instance, biological sequence and genomic feature information is generally textual, e.g. in FASTQ, BED and SRA formats. Such data can be directly fed to the transformer by word embedding or character embedding techniques (Chen *et al.*, 2022b; Ji *et al.*, 2021; Rives *et al.*, 2021); patient visit information (including disease, medication and clinical records) is represented as sequences of codes, such as EHR, ICD, where the code sequences are mapped to vector sequences in the application (Li *et al.*, 2020; Meng *et al.*, 2021; Rasmy *et al.*, 2021); the biomedical field involves images that are generally reshaped into sequences of patches for tokenization and mapped into a latent space using a trainable linear projection (Chen *et al.*, 2021a, b).

Furthermore, much more attention should be paid to multimodal learning (MML). Recently, the studies of MML with Transformer have made great progress in the field of NLP and computer vision (Chen *et al.*, 2020; Lu *et al.*, 2019; Zheng *et al.*, 2021). Since Transformer can work in a mode-independent manner, it can extract and related information from multimodal data by fusion (or alignment) of the input token embeddings of self-attention (Radford *et al.*, 2021; Xu *et al.*, 2022). Making use of biomedical codes, medical images, waveforms and genomics in pre-training models would be beneficial but requires in-depth studies of multimodal transformers.

### 4.2 Computational expense

The large amount of high-throughput sequencing data has led to the fact that many labs currently spend more on storage and computation, and the calculation and mining of massive amounts of data have become a major bottleneck for downstream studies. The powerful performance of the transformer comes largely from self-attention, which leads to the huge computational expense and makes transformer unable to model long sequences. Many efforts have been made to improve the transformer for this problem:

1. Improvements based on recursive connection: Transformer-XL (Dai *et al.*, 2019) proposed segment-level RNN mechanism and relative positional encoding to model long-distance dependence.
2. Improvements based on sparse attention: For example, Longformer (Beltagy *et al.*, 2020) proposed sliding windows, dilated sliding windows and global attention strategies to reduce the complexity of the model; Big Bird (Zaheer *et al.*, 2020) added random attention and introduced prior knowledge to limit the scope of attention and enhance efficiency; Reformer (Kitaev *et al.*, 2020) computed the *Q* and *K* matrices using the same linear layer parameters and calculated the attention score separately for each query, changing the storage expense to the square root level of the original.
3. Improvements based on low-rank decomposition: Linformer (Wang *et al.*, 2020) proposed singular value decomposition of the calculated attention matrix to transform the complexity from square to linear.
4. Improvements based on linear attention: Such as Linear Transformer (Katharopoulos *et al.*, 2020) and Performer (Choromanski *et al.*, 2021) replaced softmax with other mappings, making the multiplication complexity of *Q*, *K* and *V* matrices O(N).

In addition, Zhang *et al.* (2020) proposed Scale-dot Product Attention for dimensionality in TensorCoder, which reduced the computational expense from $O(N^{2d})$ to $O({Nd}^{2})$. When the sequence length (N) is greater than the word vector dimension (d), it can reduce the costs. Given the increasing volume of data and the complexity of analysis, developing more efficient transformer models and architectures will be another crucial direction not only for machine learning but also for bioinformatics research.

### 4.3 Model interpretability

A common criticism of deep learning models is their lack of interpretability. However, the model interpretability analysis is particularly vital when the dimension of original features is too high. Especially in the field of bioinformatics, gaining insight from the model is critical since having an interpretable model of a biological system may lead to hypotheses that can be validated experimentally. The self-attention mechanism in Transformer has notable advantages in this direction. For example, through the analysis of attention maps, DNABERT (Ji *et al.*, 2021) could visualize important areas that contributed to model decision-making, thereby improving the interpretability of the model. Expect for prediction, DNABERT could directly rank the importance of the input nucleotide molecules and analyze the relationship between the input sequence contexts, resulting in better visualization information and accurate motifs extraction. Most of the attention heads of the Transformer-XL-based network architecture (Clauwaert *et al.*, 2021) could successfully identify and characterize transcription factors’ binding sites and consensus sequences, which showed that transformer has unique potential for genome annotation tasks and biological significance extraction. Reflecting the contribution of each gene and the interaction between gene pairs by self-attention mechanism, scBERT (Yang *et al.*, 2022) can obtain the top attention genes corresponding to a specific cell type, which is important for cell type annotation. The attention mechanism in DeepMAPS enhanced biological interpretability by fully capturing complex molecular mechanisms and cellular heterogeneity (Ma *et al.*, 2021). And the attention of GTN could identify potential miRNA-mRNA targeting pathways and biomarkers, which is not easy or even impossible to be achieved by other models (Kaczmarek *et al.*, 2021). Interpretability makes the model itself, rather than results or data, become the source of knowledge. How to better utilize the self-attention mechanisms to demonstrate the biological insight behind the models will become one of the most desirable improvements in transformer-based applications in bioinformatics.

## 5 Conclusion

The recent development of transformer-based language models has substantially enriched the NLP field with novel architectures of self-attention that can greatly improve model accuracy, efficiency and interpretability. As a new potential force, transformer-based models have brushed up on SOTA performance with a large margin in most bioinformatics tasks. For example, the precision of GeneBERT in promoter classification, TFBS classification and disease risks estimation tasks was 0.130, 0.674 and 0.510 higher than that of the second-best method, respectively; the accuracy of scBERT in the prediction of novel and known cell types increased by 0.155 and 0.158, respectively; ESM-1b increased precision on secondary structure and contact predictions by 0.092 and 0.279; InferBERT almost doubled the number of identified causal factors on acute liver failure (from 23 to 43). Although several models did not reach SOTA in terms of evaluation metrics, such as GTN and ChemBERTa, they also made significant breakthroughs, and they were still innovative for other properties, such as the robustness to high-dimensional, small sample size and heterogeneous data.

Nevertheless, the development and application of transformers in bioinformatics are still in their infancy. There are many directions for further exploration, such as developing better pre-training methods, improving model flexibility, standardizing benchmarks and mitigating bias. Research in these directions will improve the analysis and interpretation of transformer-based models, and help the research community to utilize various biological data effectively. We hope this review article sparks thoughts on transformer-based language models across multiple disciplines and will inspire future research and applications that revolutionize biological and biomedical research and open up new avenues for the diagnosis and treatment of human diseases.

## Supplementary Material

vbad001_Supplementary_Data
