# Supplementary material for: Applications of transformer-based language models in bioinformatics: a survey
Source: Bioinform Adv. 2023 Jan 11;3(1):vbad001. doi: 10.1093/bioadv/vbad001 (PMC9950855; doi:10.1093/bioadv/vbad001)
Supplement: vbad001_Supplementary_Data [file vbad001_supplementary_data.docx]

Supplementary Information for

“Applications of Transformer-based Language Models in Bioinformatics: A Survey”

Shuang Zhang^1^, Rui Fan^1^, Yuti Liu^1^, Shuang Chen^1^, Qiao Liu^2^ and Wanwen Zeng^2,*^

*^1^College of Software, Nankai University, Tianjin, 300350, China and ^2^Department of Statistics, Stanford University, Stanford, CA 94305, USA.*

**Supplementary Table S1.** Overview of the abbreviations in this paper, sorted alphabetically.

| Abbreviation | Definition |
| --- | --- |
| auPRC | Area under the Precision-Recall Curve |
| Bi-RNN | Bi-directional RNN |
| CBOW | Continuous Bag of Words Model |
| CNN | Convolutional neural network |
| DEGs | Differentially expressed genes |
| DTI | Drug-target interaction |
| EHRs | Electronic health records |
| EPI | Enhancer-promoter interaction |
| FFN | Feed-Forward Network |
| GCN | Graph Convolutional Network |
| GO | Gene ontology |
| GRU | Gate recurrent unit |
| GTN | Graph transformer network |
| ICD | International classification of diseases |
| LSTM | Long Short-Term Memory |
| m7G | N7-methylguanosine |
| MCC | Matthews Correlation Coefficient |
| miRNA | microRNA |
| MLP | Multi-Layer Perceptron |
| mRNA | Messenger RNA |
| MSA | Multiple sequence alignment |
| NER | Named Entity Recognition |
| NGS | Next-generation sequencing |
| NLP | Natural language processing |
| PCP | Protein contact prediction |
| QA | Question Answering |
| RE | Relationship Extraction |
| RNN | Recurrent Neural Network |
| ROC-AUC | Area under the Receiver operating characteristic curve |
| scRNA-seq | Single-cell RNA-sequencing |
| SGAs | Somatic genomic alterations |
| SOTA | State-of-the-art |
| SVM | **Support Vector Machine** |
| TFBS | Transcription factor binding site |
| TSS | Transcription start site |
| ViT | Vision Transformer |
